# Hugan Qingzhi tablets attenuates endoplasmic reticulum stress in nonalcoholic fatty liver disease rats by regulating PERK and ATF6 pathways

**DOI:** 10.1186/s12906-024-04336-1

**Published:** 2024-01-12

**Authors:** Miaoting Yang, Xiaorui Yao, Fan Xia, Shijian Xiang, Waijiao Tang, Benjie Zhou

**Affiliations:** 1 Department of Pharmacy, People’s Hospital of Longhua, Shenzhen, 518109 Guangdong China; 2 Department of Pharmacy, Shantou Central Hospital, Shantou, 515041 Guangdong China; 3 Department of Pharmacy, the Seventh Affiliated Hospital, Sun Yat-sen University, Shenzhen, 518107 PR China; 4 Zhujiang Hospital, Southern Medical University, Guangzhou, 510282 Guangdong China

**Keywords:** Chinese traditional medicine, Hugan Qingzhi tablets (HQT), Non-alcoholic fatty liver disease (NAFLD), Hepatoprotective effect, Endoplasmic reticulum stress (ERS) pathway

## Abstract

**Background:**

Endoplasmic reticulum (ER) stress, promoting lipid metabolism disorders and steatohepatitis, contributes significantly to the pathogenesis of nonalcoholic fatty liver disease (NAFLD). Hugan Qingzhi tablets (HQT) has a definite effect in the clinical treatment of NAFLD patients, but its mechanism is still unclear. This study aims to investigate the effects of HQT on ER stress in the liver tissues of NAFLD rats and explore the underlying mechanism.

**Methods:**

The NAFLD rat model was managed with high-fat diet (HFD) for 12weeks. HQT was administrated in a daily basis to the HFD groups. Biochemical markers, pro-inflammatory cytokines, liver histology were assayed to evaluate HQT effects in HFD-induced NAFLD rats. Furthermore, the expression of ER stress-related signal molecules including glucose regulating protein 78 (GRP78), protein kinase RNA-like endoplasmic reticulum kinase (PERK), p-PERK, eukaryotic translation initiation factor 2α (EIF2α), p-EIF2α, activating transcription factor 4 (ATF4), acetyl-coenzyme A-carboxylase (ACC), activating transcription factor (ATF6), and nuclear factor-kappa B-p65 (NF-κB-p65) were detected by western blot and/or qRT-PCR.

**Results:**

The histopathological characteristics and biochemical data indicated that HQT exhibited protective effects on HFD-induced NAFLD rats. Furthermore, it caused significant reduction in the expression of ERS markers, such as GRP78, PERK, p-PERK, and ATF6, and subsequently downregulated the expression of EIF2α, p-EIF2α ATF4, ACC, and NF-κB-p65.

**Conclusions:**

The results suggested that HQT has protective effect against hepatic steatosis and inflammation in NAFLD rats by attenuating ER stress, and the potential mechanism is through inhibition of PERK and ATF6 pathways.

**Supplementary Information:**

The online version contains supplementary material available at 10.1186/s12906-024-04336-1.

## Background

Nonalcoholic fatty liver disease (NAFLD) is a clinicopathological disease, which is defined as an excessive accumulation of fat in the hepatocyte cytoplasm and the formation of lipid droplets, associated with hepatomegaly and inflammation [1, 2]. It ranges from simple steatosis to steatohepatitis, cirrhosis, and could eventually lead to hepatocellular carcinoma (HCC). With dramatic changes in lifestyles over the last 20 years, NAFLD has become the most prevalent liver disorder in China [3], and its potential risks are gradually recognized, thus the research related to NAFLD disease is attracting a growing attention. Currently, it has become clear that NALFD is a multifactorial disease closely associated with hepatic steatosis, insulin resistance, inflammatory response, oxidative stress, etc [4]. Sustained endoplasmic reticulum stress (ERS) is involved in the regulation of the above physiological changes and plays an important role in the development of NALFD [5–7].

The ER is the main site of intracellular protein synthesis and modification. When the synthesis of protein precursors exceeds the quality control capacity of the ER, it will cause unfolded protein response (UPR), and then induce ER Stress, resulting in hepatic lipid metabolism disorder, inflammation and hepatocyte apoptosis [8]. Mounting evidence indicates that ER stress-induced activation of protein kinase RNA-like endoplasmic reticulum kinase (PERK) pathway can up-regulate the expression of lipogenesis-related genes such as acetyl-coenzyme A-carboxylase (ACC), thereby inducing excessive accumulation of hepatic lipids [9–11]. Moreover, ER stress activates the activating transcription factor 6 (ATF6) pathway to upregulate nuclear factor-kappa B (NF-κB) expression, exacerbating the hepatic inflammatory response and leading to liver steatosis to non-alcoholic steatohepatitis (NASH) [12, 13]. Therefore, PERK and ATF6 pathways have emerged as particularly promising therapeutic targets for the prevention of fatty liver disease.

Hugan Qingzhi tablet (HQT), a traditional Chinese medicine formula, has a long history of use in alleviating NAFLD in clinical practice. The formula consists of five herbal medicines, including *Rhizoma alismatis, Fructus crataegi, Pollen typhae, Folium nelumbinis* and *Radix notoginseng*. Our previous studies showed that HQT had anti-inflammatory and lipid-lowering effects on NAFLD [14–16]. However, the mechanisms associated with its improvement in NAFLD need to be further investigated. In our proteomics studies based on isobaric tags for relative and absolute quantitation of NAFLD rat livers, Kyoto Encyclopedia of Genes and Genomes (KEGG) pathway enrichment analysis indicated that the ER stress is closely related to the pathogenesis of NAFLD [17]. In vivo studies show that HQT-medicated serum could effectively alleviate the ERS state of L02 cells [18]. However, immediate evidence for the effect of HQT on the regulation of the ER stress is still absent in vivo. Therefore, the aim of this study was to investigate whether HQT protects against NAFLD by attenuating ER stress through PERK and ATF6 signaling pathways in rats.

## Methods

### Preparation of HQT

HQT was produced by Zhujiang Hospital, Southern Medical University, Guangzhou, China. It was prepared as follows: firstly, mixtures of 30% *Fructus crataegi*, 30% *Rhizoma alismatis*, 20% *Folium nelumbinis*, and 15% *Pollen typhae* were infiltrated with 70% ethanol (6:1, v/w) for 1.5 h and then extracted by reflux extraction for 2 h. The process was repeated three times. The yield of dried extract from the starting crude was 14.45% (w/w). After that, *5% Radix notoginseng* was ground, sieved, and added to the dried extract to prepare HQT. The identification and quantification of the main components of HQT have been reported in our previous studies [19–21].

### Animals and experimental design

Sixty SPF-grade male Sprague-Dawley rats (180-220 g) were obtained from Experimental Animal Center of Southern Medical University (Guangzhou, China, quality certificate number: SCXK (Yue) 20,110,015). Animal experiments were approved by the Animal Ethics Committee of the Southern Medical University (Animal Welfare Assurance L2016133), with reference to the Guidelines for the Care and Use of Laboratory Animals in China. All efforts were made to minimize the suffering of the animals involved in the experiments, such as provide a clean and comfortable environment with adequate food, drinking water, and space; perform animal experiments with standardized practices; anesthetize adequately with pentobarbital prior to obtaining blood samples; euthanasia by means of cervical dislocation and ensuring that tissue samples are taken after the death of the animal, etc. Rats were raised at 20–25℃, subjected to a 12-hour light-dark cycle in a pathogen-free laboratory, and allowed to drink and eat freely. After one-week of conditioning, 60 rats were randomly assigned to 6 groups (*n* = 10 per group). Rats in the control group were given 1mL/100 g of distilled water and standard chow diet (SCD); rats in the model group were given distilled water and high-fat diet (HFD, ingredient: 20% sucrose, 15% lard, 1.2% cholesterol, 0.2% sodium cholic acid, 10% casein, 0.6% calcium hydrogen phosphate, 0.4% stone powder, 0.4% premix and 52.2% base feed); rats in the fenofibrate (a commonly used lipid-lowering drug and used as positive control drug) group were orally administered 0.1 g/kg fenofibrate suspension (FF) and HFD; rats in the other three groups were given HFD + HQT suspension in a low (HL, 0.54 g/kg), moderate (HM, 1.08 g/kg), or high dosage (HH, 2.16 g/kg). The formulation of HFD has been described in previous studies [19–21]. After 12 weeks of treatment, rats were anesthetized with 2% pentobarbital sodium (3 ml/kg body weight) and blood was collected through the abdominal aorta.

### Serum biochemical analysis

Serum alanine aminotransferase (ALT), aspartate aminotransferase (AST), high-density lipoprotein cholesterol (HDL-C), low-density lipoprotein cholesterol (LDL-C), triglyceride (TG), and total cholesterol (TC) were assessed by automatic biochemical analyzer (Olympus AU5400, Tokyo, Japan).

### Pro-inflammatory cytokine assays

Markers of inflammation, including the interleukin-1β (IL-1β), interleukin-6 (IL-6) and tumor necrosis factorα(TNF-α), were measured in liver homogenates using an ELISA kit (Multisciences, Hangzhou, China) according to the manufacturer’s instructions [22].

### Liver histological examination

To better visualize the accumulation of lipids in liver tissue, the right lobe of liver tissue was weighed and fixed with 4% paraformaldelyde solution for liver histological examination. Then the liver tissues were paraffin-embedded, cut into 5 μm-thick sections, and stained with H&E (hematoxylin-eosin). In addition, frozen sections of liver tissues (10 μm per section) were stained with Oil Red O-Thoumarin (Sigma-Aldrich, USA). The stained sections were photographed using an Olympus light microscope (Olympus Corporation, Tokyo, Japan) at a magnification of 400×. For transmission electron microscopy (TEM), hepatic tissues were collected, fixed in 2.5% glutaraldehyde and post-fixed with 1% phosphate-buffered osmium tetroxide. Then, liver tissues were embedded, sliced (60-70 nm), and double-stained. Images were acquired using transmission electron microscopy. Histopathological analysis was performed in six randomly selected fields from each Sects. [23, 24].

### Quantitative real-time PCR (qRT-PCR) analysis

Liver tissue samples were quickly ground in liquid nitrogen and total RNA was extracted by adding Trizol Reagent (Invitrogen, USA) according the manufacturer’s instructions. The RNA was reverse-transcribed into cDNA by using the PrimeScript® RT kit (Takara, Japan) at 42 °C for 45 min, followed by incubation at 95 °C for 5 min. qRT-PCR was used to measure the mRNA expression levels of GRP78, ATF4, ACC, ATF6, and β-ACTIN. For real-time PCR, the cDNA and primers were prepared with a SYBR®Premix Ex TaqTMII Kit (Takara, Japan), according to the instruction manual. The amplification conditions were: initial denaturation (95℃, 30s, 1cycle); amplification (95℃, 5s, 60℃, 34s, 40cycles); dissolution curve (95℃, 5s, 60℃, 1 min, 1cycle) [25]. The sequences of the primers are shown in Table 1.

**Table 1:** Primer design for rtPCR

Gene	Forward	Reverse
GRP78	AACCCAGATGAGGCTGTAGCA	ACATCAAGCAGAACCAGGTCAC
ATF4	TGGTCTCAGACAACAGCAAG	AGCTCATCTGGCATGGTTTC
ATF6	TATCCCTCCACCTCCATGTCA	TCTCGATTTGGTCCTTTCCACT
ACC	ATGAAGGCTGTGGTGATGGA	TGGTGGTCTTGCTGAGTTGA
β-ACTIN	CCCATCTATGAGGGTTACGC	TTTAATGTCACGCACGATTTC

### Western blotting analysis

**Fig. 1:**
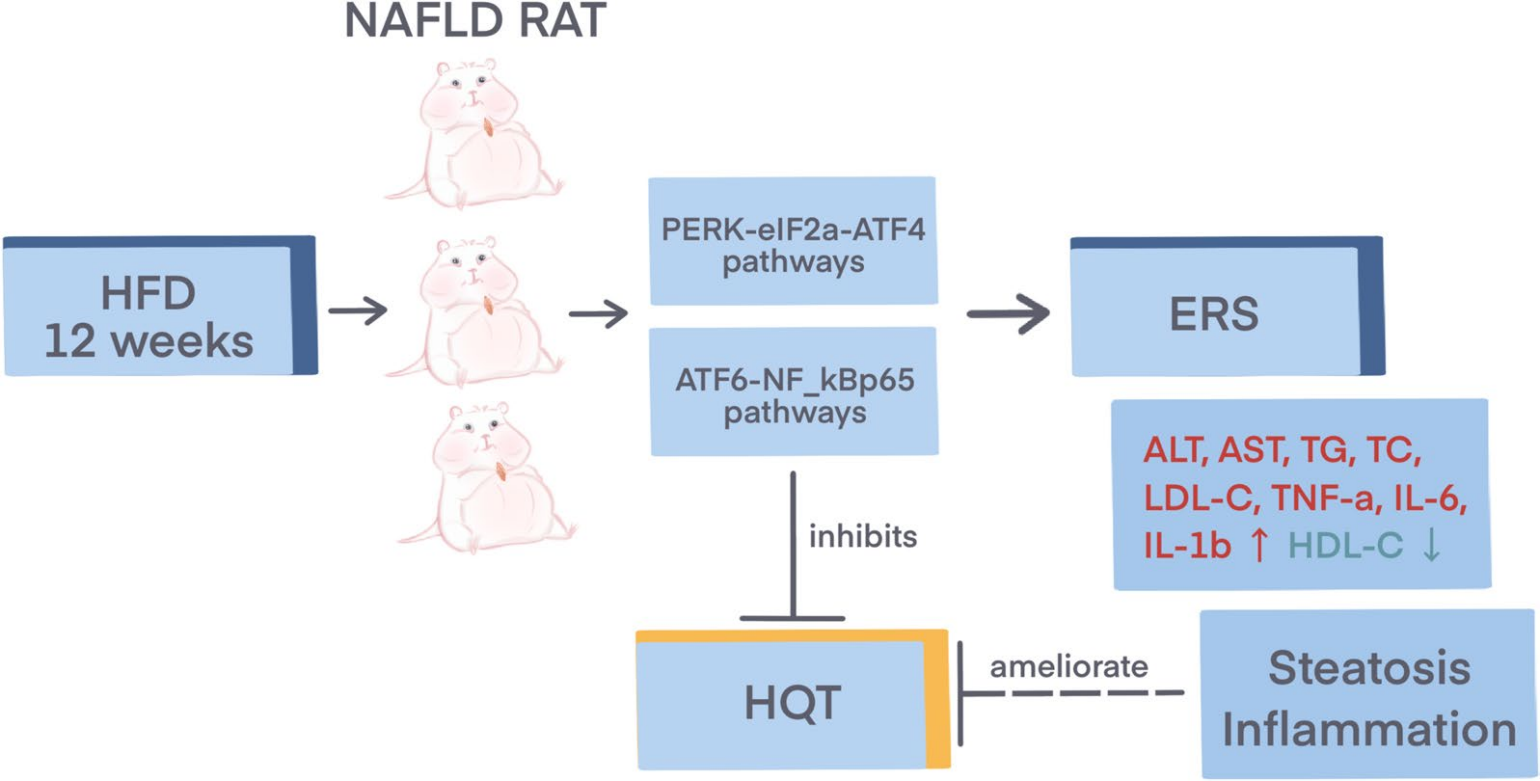
Hugan Qingzhi tablets attenuates endoplasmic reticulum stress in nonalcoholic fatty liver disease rats by regulating PERK and ATF6 pathways. ↑: up-regulation; ↓: down-regulation

The liver tissues were homogenized with RIPA lysis buffer and the total protein content in the supernatant of each sample was determined by BCA protein assay (Beyotime Biotech, China) according to the manufacturer’s instructions. The protein samples were separated by electrophoresis in 15% SDS-PAGE gel and then transferred to a polyvinylidene fluoride (PVDF) membrane, which was cut into different bands according to the molecular weight of the protein. The membranes were blocked with 5% non-fat milk for 1 h at room temperature and then incubated with primary antibodies (GRP78, 1: 3000, Abcam; PERK, 1: 1000, Abcam; p-PERK, 1: 1000, Beyotime; ATF4, 1: 1000, Beyotime; ACC, 1:1000, Beyotime; ATF6, 1: 1000, Beyotime; EIF2α, 1: 1000, Beyotime; p-EIF2α, 1: 1000, Beyotime; NF-κB-p65, 1: 500, Abcam; GAPDH, 1: 1000, Santa Cruz; Histone H3, 1: 3000, Santa Cruz) at 4℃ overnight. Subsequently, the membrane was washed with PBS and incubated with peroxidase-conjugated secondary antibodies at room temperature for 1 h [26]. Protein bands were visualized using enhanced chemiluminescence detection reagent (Sigma Chemical Co., USA) and analyzed with quantitative image analysis software (Fig. 1).

### Statistical analysis

All data were expressed as mean ± standard deviation. Statistically significant differences between groups were evaluated using Student’s t-test or one-way analysis of variance (ANOVA) using the SPSS 23.0 (IBM Corporation, NY, USA) statistical package. Differences were considered statistically significant when the *p* value was less than 0.05.

## Results

### Effect of HQT on body weight and liver index in rats

**Fig. 2:**
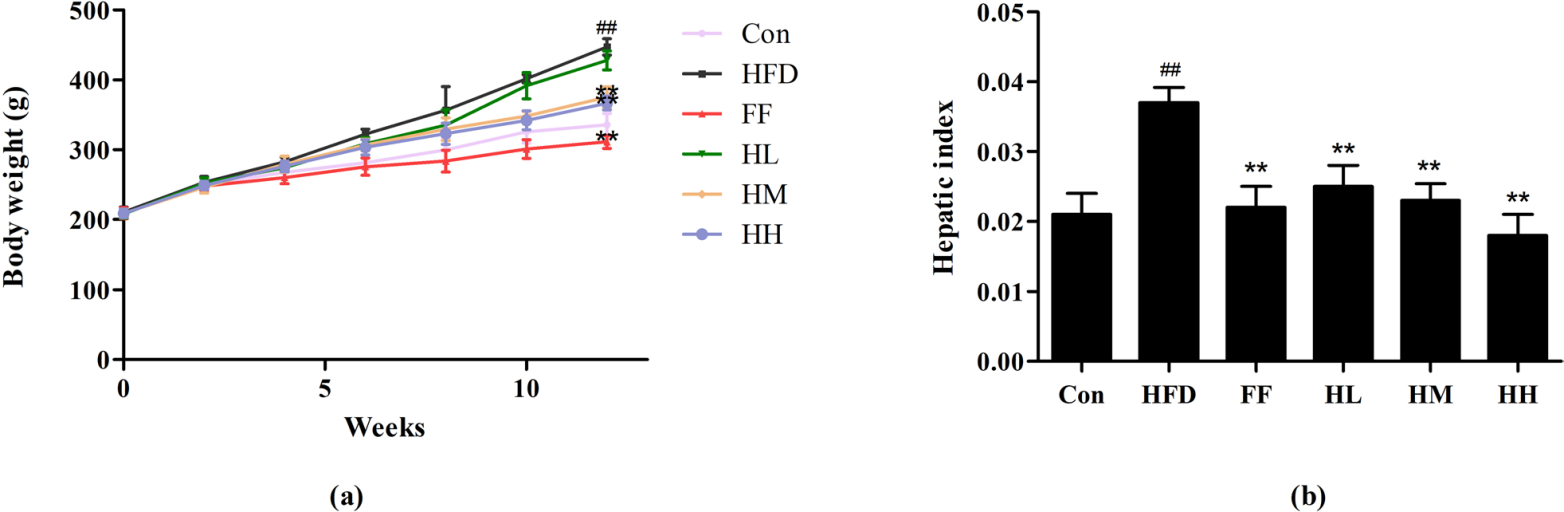
Rat body weight (**a**) and hepatic index (**b**) changes. Con: control group; HFD: high-fat diet group; FF: HFD + fenofibrate group; HL: HFD + HQT low dosage group; HM: HFD + HQT moderate dosage group; HH: HFD + HQT high dosage group. ^*^ *p* < 0.05, ^**^ *p* < 0.01 versus Con group. ^#^ *p* < 0.05, ^##^ *p* < 0.01 versus HFD group

After one week of acclimatization feeding, there was no statistically significant difference in the body weights of the rats in each group. After 12 weeks, the body weights of rats in the HFD group increased significantly (*p* < 0.01) compared to the Con group, whereas the body weights of the rats in the HM, HH and FF groups decreased (*p* < 0.01) compared to the HFD group. In terms of liver index, the HFD group showed a remarkable increase in liver index compared to the Con group (*p* < 0.001), while the HL, HM, HH, and the FF group showed a decrease in liver index compared to the HFD group (*p* < 0.01), as shown in Fig. 2.

### Effects of HQT on hepatic function and serum lipids

**Fig. 3:**
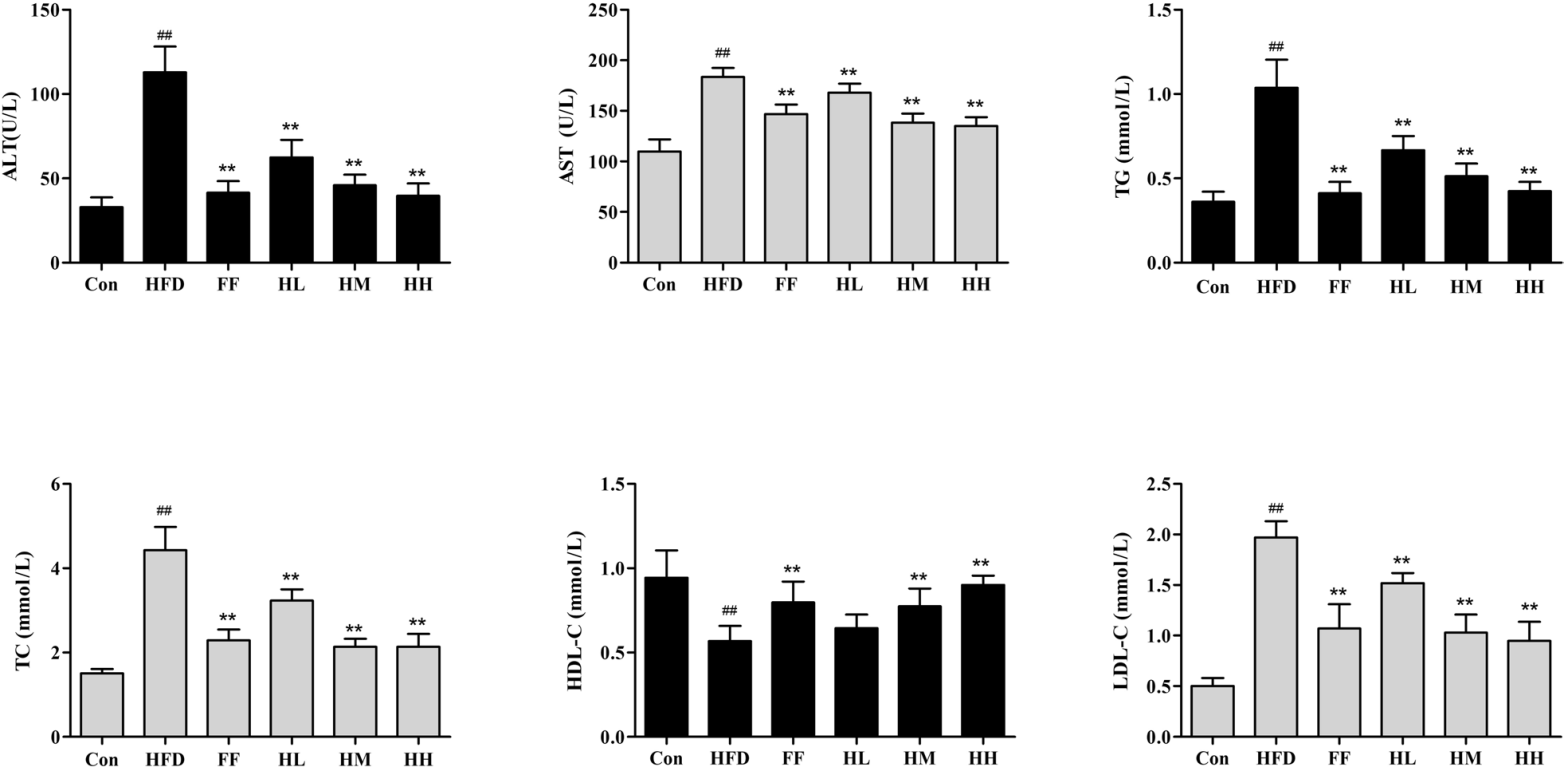
Effects of HQT on the serum levels of ALT, AST, TG, TC, LDL-C and HDL-C in NAFLD rats. Con: control group; HFD: high-fat diet group; FF: HFD + fenofibrate group; HL: HFD + HQT low dosage group; HM: HFD + HQT moderate dosage group; HH: HFD + HQT high dosage group. ^*^ *p* < 0.05, ^**^ *p* < 0.01 versus Con group. ^#^ *p* < 0.05, ^##^ *p* < 0.01 versus HFD group

In order to evaluate whether HQT has a protective effect on high fat diet injured liver, the study measured serum lipids and hepatic function-related indexes in rats. Compared with the Con group, the serum ALT and AST levels of rats in the HFD group were significantly increased (*p* < 0.01), compared with the HFD group, HM, HL and fenofibrate treated rats showed a significant decrease in the levels of serum ALT and AST (*P* < 0.01). Meanwhile, compared with Con group, the levels of serum TG, TC and VDL-C were significantly increased (*p* < 0.01) and HDL-C levels were significantly decreased (*p* < 0.01) in HFD group; after medium and high doses of HQT treatment, serum TG, TC, HDL-C and LDL-C levels were reversed compared with the HFD group *(p* < 0.01), as shown in Fig. 3.

### Effect of HQT on inflammatory cytokine levels in hepatic tissues

**Fig. 4:**
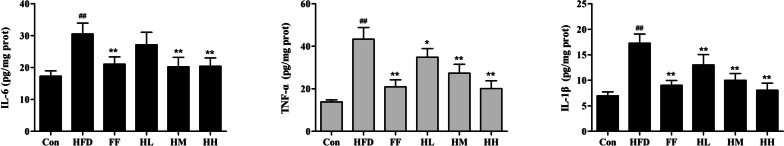
Effects of HQT on the levels of TNF-α, IL-6 and IL-1β in NAFLD rat livers. Con: control group; HFD: high-fat diet group; FF: HFD + fenofibrate group; HL: HFD + HQT low dosage group; HM: HFD + HQT moderate dosage group; HH: HFD + HQT high dosage group. ^*^ *p* < 0.05, ^**^ *p* < 0.01 versus Con group. ^#^ *p* < 0.05, ^##^ *p* < 0.01 versus HFD group

The inflammatory response is an important component of the pathogenesis of NAFLD. Compared with the Con group, the levels of TNF-α, IL-6 and IL-1β in the liver tissue homogenates of rats in the HFD group were statistically increased (*p* < 0.01). In contrast, HM, HH and FF groups treatment successfully restored (*p* < 0.01) the expression of these inflammatory cytokines in NAFLD rats (Fig. 4).

### Analysis of hepatic histopathology

**Fig. 5:**
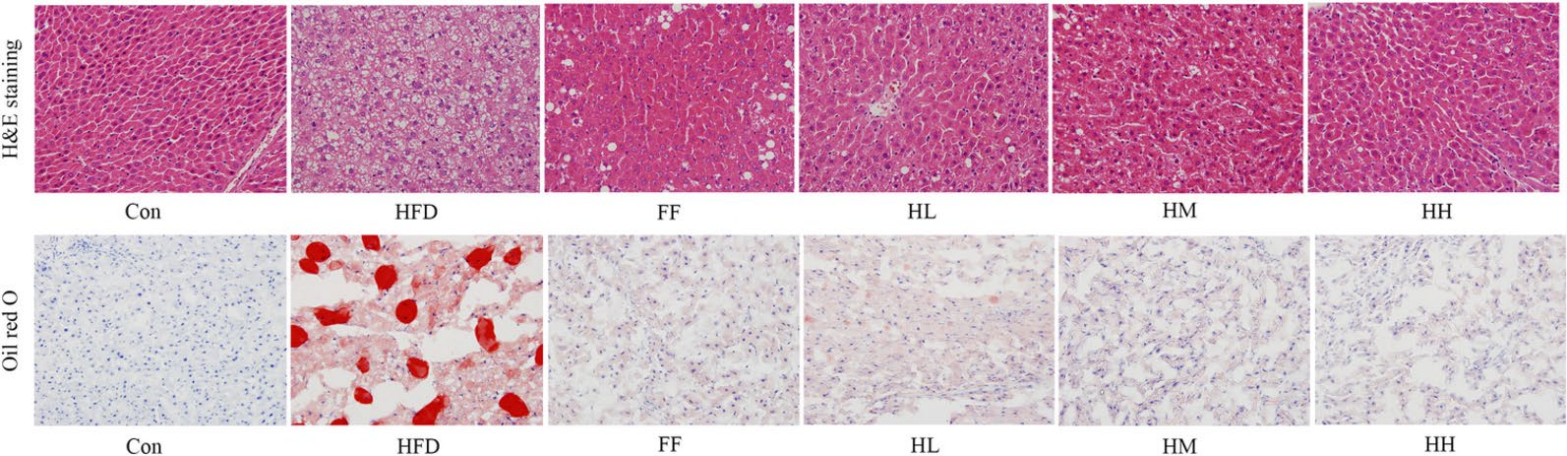
Effects of HQT on histopathological examination by H&E and Oil Red O (400× magnification). Con: control group; HFD: high-fat diet group; FF: HFD + fenofibrate group; HL: HFD + HQT low dosage group; HM: HFD + HQT moderate dosage group; HH: HFD + HQT high dosage group

The photomicrographs of the H&E-stained liver sections are shown in Fig. 5. In the control group, the hepatocytes were neatly arranged, without steatosis, with clear lobular structure and no inflammatory cell infiltration in the portal area; in the HFD group, the hepatocytes were swollen, with lipid droplet vacuoles of different sizes, marginalized nuclei and inflammatory cell infiltration in the portal area; in the HL group, the hepatocytes were improved but not significantly compared with the HFD group; in the HM, HH and FF groups, the steatosis was significantly alleviated and the inflammatory cells were reduced compared with the HFD group. Oil red O staining demonstrated that there was no obvious steatosis in hepatocytes of the control group, and a large number of red-stained lipid droplets appeared in the hepatocytes of the HFD group; the hepatocytes of the HL group improved compared with the model group, and the amount of lipid droplets tended to decrease; there were sporadic lipid droplets in the hepatocytes of the HM, HH and fenofibrate groups, and steatosis was significantly alleviated. The results showed that HFD-induced hepatocyte steatosis in rats and HQT could ameliorate the lipid accumulation in liver.

**Fig. 6:**
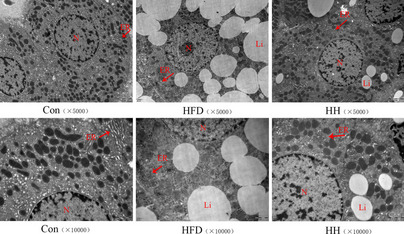
Transmission electron microscopy of the ER in each group of hepatocytes (5000× magnification, 10,000× magnification). Li: lipid, M: mitochondria, Er: endoplasmic reticulum. Con: control group; HFD: high-fat diet group; HH: HFD + HQT high dosage group

The endoplasmic reticulum substructure of hepatocytes was visualized using transmission electron microscopy (Fig. 6). The endoplasmic reticulum of the control group was neatly arranged and flattened in the shape of a vesicle, and the ribosomes were tightly attached to the outer flat pool membrane, and no obvious lipid vesicles were seen in the cytoplasm. In contrast, the endoplasmic reticulum structure of the HFD group showed severe damage, as evidenced by rough endoplasmic reticulum dilatation and degranulation. And the lipid droplet vacuoles of different sizes in the cytoplasm disrupt the distribution and structure of the endoplasmic reticulum. While, after the high-dose HQT intervention, the overall cell structure tended to be normalized and the lipid vacuolation phenomenon was significantly reduced, and the structure and morphology of endoplasmic reticulum were significantly.

### Effect of HQT on PERK pathway proteins and mRNA of endoplasmic reticulum stress

**Fig. 7:**
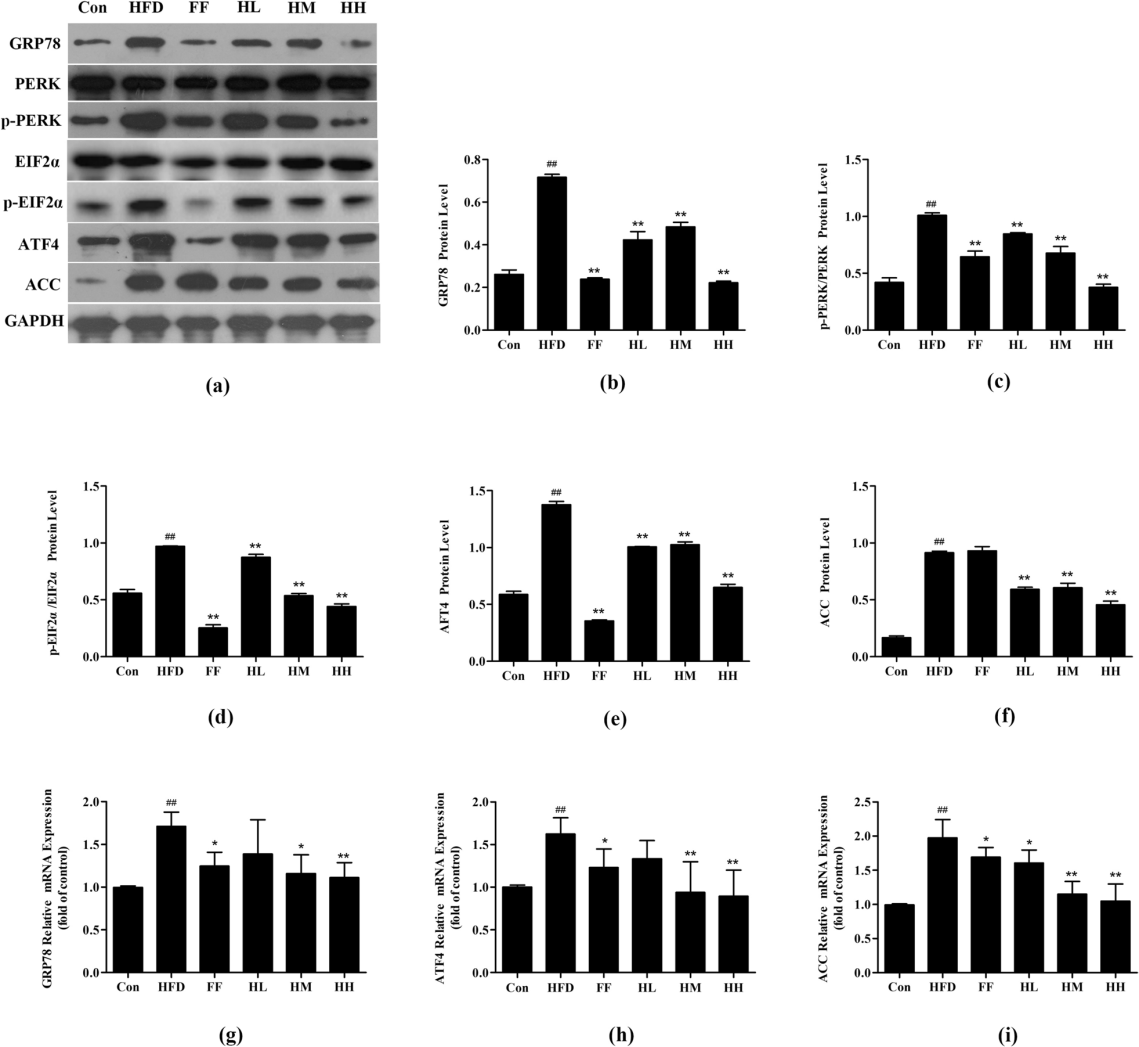
Effect of HQT on PERK pathway proteins and mRNA of ERS. **a-f** The expression levels of GRP78, PERK, p-PERK, EIF2α, p-EIF2α, ATF4 and ACC protein in rat liver tissues. **g-i** The expression levels of GRP78, ATF6 and ACC mRNA in rat liver tissues. Con: control group; HFD: high-fat diet group; FF: HFD + fenofibrate group; HL: HFD + HQT low dosage group; HM: HFD + HQT moderate dosage group; HH: HFD + HQT high dosage group. ^*^ *p* < 0.05, ^**^ *p* < 0.01 versus Con group. ^#^ *p* < 0.05, ^##^ *p* < 0.01 versus HFD group

The expression of PRRK pathway-related proteins and mRNA were examined by western blot and RT-PCR. Compared with the Con group, the expression of GRP78, a marker protein of endoplasmic reticulum stress, was significantly increased in the HFD group (*p* < 0.01). HQT treatment was able to dose-dependently down-regulate the expression of HFD-induced GRP78 (*p* < 0.01). Meanwhile, the protein expression levels of p-PERK/PERK, p-EIF2α, ATF4, and ACC in the HFD group were significantly higher than those in the Con group (*p* < 0.01), whereas the protein expression levels of p-PERK/PERK, p-EIF2α, ATF4, and ACC in the HL, HM and HH groups were all downregulated (*p* < 0.01), and the HH and FF groups gradually converged to the expression levels of the Con group. The expression of GRP78, ATF4, and ACC mRNA showed similar results (Fig. 7).

### Effect of HQT on ATF6 pathway proteins and mRNA of endoplasmic reticulum stress

**Fig. 8:**
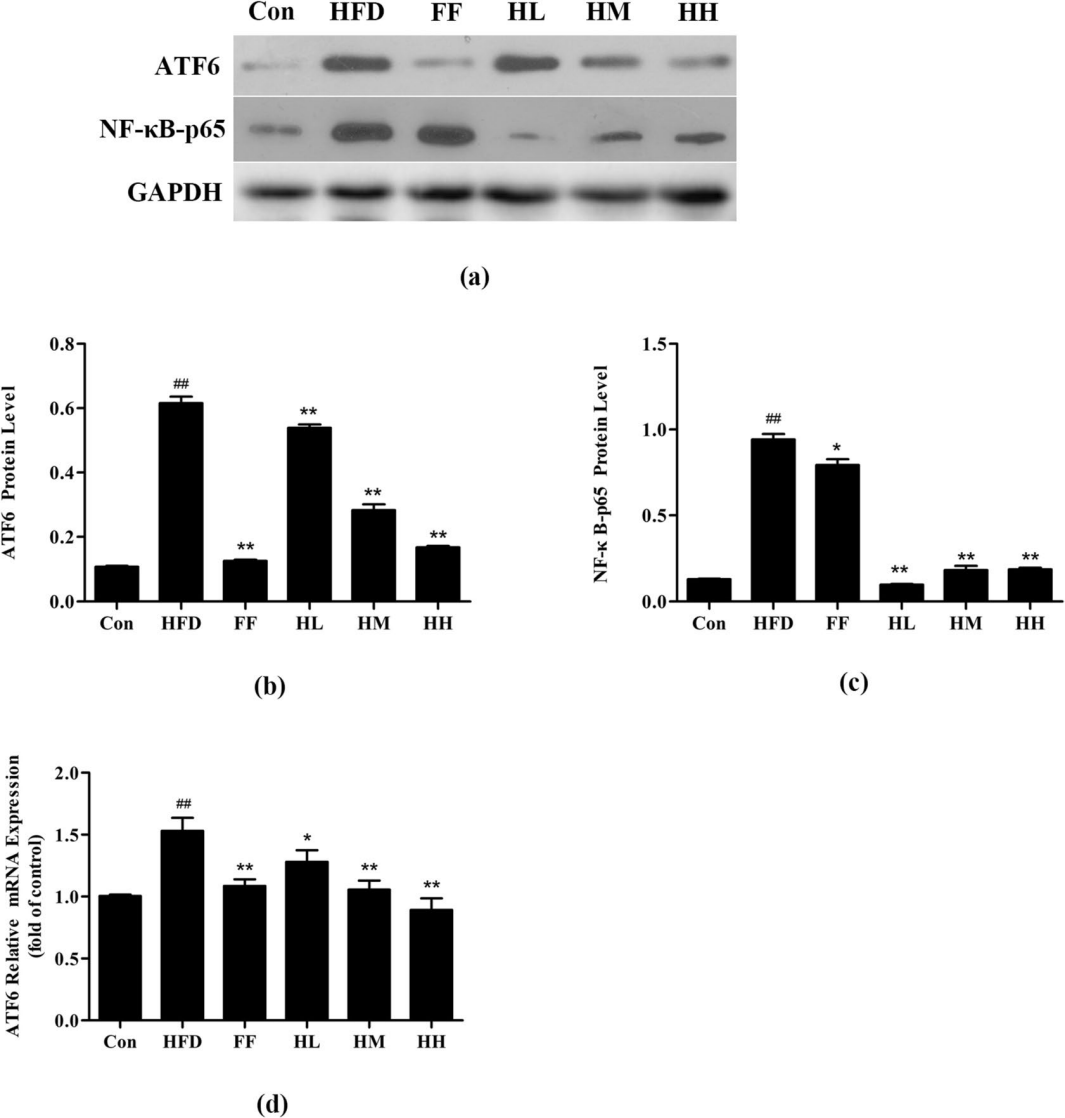
Effect of HQT on ATF6 pathway proteins and mRNA of ERS. **a-c** The expression levels of ATF6 and NF-κB-p65 protein in rat liver tissues. **d** The expression levels of ATF6 mRNA in rat liver tissues. Con: control group; HFD: high-fat diet group; FF: HFD + fenofibrate group; HL: HFD + HQT low dosage group; HM: HFD + HQT moderate dosage group; HH: HFD + HQT high dosage group. ^*^ *p* < 0.05, ^**^ *p* < 0.01 versus Con group. ^#^ *p* < 0.05, ^##^ *p <* 0.01 versus HFD group

ATF6 is one of the marker signaling molecules of endoplasmic reticulum stress. As shown in Fig. 8, the expression of ATF6 in the HFD group was much higher than that in the Con group (*p* < 0.01), while the expression level of ATF6 in HM, HH and FF groups were all downregulated. In addition, NF-κB-p65 protein expression was significantly upregulated in the HFD group compared with the Con group (*p* < 0.01), whereas NF-κB-p65 expression showed decrease markedly after HQT treatment (*p* < 0.01). The expression of ATF6 mRNA showed similar results.

## Discussion

With the gradual westernization of the dietary spectrum in China in recent years, the prevalence of NAFLD in the Chinese population has increased significantly to 15%, becoming the second most common liver disease after viral hepatitis [27]. A long-term high-fat diet impairs liver function, leads to liver steatosis and can induce NASH, which can eventually lead to hepatic fibrosis and even hepatocellular carcinoma [28]. The pathogenesis of NAFLD is very complex. In addition to the classical " second hit” theory, the role of endoplasmic reticulum stress has been widely emphasized by scholars [6]. HQT has been identified as a potential modulator of NAFLD through its lipid-lowering and anti-inflammatory effects [14]. However, the mechanism of anti-inflammatory and hypolipidemic effects of HQT in NAFLD rats is not clear. The results of the present study showed that the body weight and liver index of the rats in the HFD group increased markedly compared with those in CON group. The serum ALT, AST, TC, LDL-C, TNF-α, IL-6, and IL-1β levels were significantly increased in rats of the HFD group than those of the Con group (*p* < 0.01), whereas HDL-C level was significantly higher than that of the Con group (*p* < 0.01). The pathological results showed that the hepatocytes were diffusely fatty and the endoplasmic reticulum was dilated into vesicles, thus indicating that the NAFLD rats were successfully modeled in this study. Meanwhile, the HM and HH groups significantly reduced serum ALT, AST, TC and LDL-C levels, increased LDL-C levels, inhibited inflammatory factors and improved liver lipid accumulation and inflammatory response in NAFLD rats, indicating that HQT can effectively alleviate the development process of NAFLD.

Recent studies demonstrated that ERS plays an important role in the development of NAFLD [29, 30]. Endoplasmic reticulum, which is abundant in hepatocytes, is an important subcellular organelle in eukaryotic cells with functions in lipid and carbohydrate synthesis, drug metabolism, storage and release of Ca^2+^ to maintain intracellular calcium homeostasis [31]. Under physiological or pathological conditions, the dynamic balance of the ER is disrupted. Misfolded proteins accumulate in ER lumen and impair normal physiological functions. This subsequently leads to ER stress, which in turn activates the cellular UPR pathway [8, 32]. In contrast, persistent or excessive ER stress causes the cell’s self-repair capacity to be insufficient to resist external stimuli, activating the endoplasmic reticulum overload response (EOR) and secondary responses such as lipid accumulation, inflammatory response, insulin resistance and apoptosis [13]. Meanwhile, high cholesterol and high TG levels in NAFLD can induce ERS, which further exacerbates cholesterol accumulation and ultimately leads to a vicious cycle of NAFLD development [33]. GRP78, a signature protein of endoplasmic reticulum stress, is a molecular chaperone protein located in the ER which is involved in protein folding and translocation to maintain endoplasmic reticulum homeostasis, thus providing protection to cells, and its expression is significantly increased in the occurrence of ERS [34]. In the present study, we first examined the expression of GRP78 gene and protein in liver tissues of NAFLD rats, and the results showed that the expression of GRP78 mRNA and protein was significantly upregulated in the HFD group rats, suggesting that HFD induced a significant ERS response. Interestingly, GRP78 expression was significantly down-regulated by HQT and FF treatment, suggesting that ERS was significantly inhibited by HQT and FF intervention.

To further explore the mechanism of the effects of HQT on ERS-related pathways, we examined the effects of HQT on UPR-related signaling pathways. Under normal physiological conditions, GRP78 binds to three membrane-spanning proteins PERK, IRE1 and ATF6 on the endoplasmic reticulum membrane, leaving it in an inactive state [32]. In pathological conditions, endoplasmic reticulum stress occurs and GRP78 is separated from the three transmembrane proteins, and unfolded proteins accumulate in the ER to compete for GRP78 binding and activate the three transmembrane proteins for ERS signaling [35, 36]. In early ERS, the PERK-mediated signaling pathway is activated initially. PERK is a type I transmembrane protein of the endoplasmic reticulum and a member of the eukaryotic translation initiation complex 2α kinase family. Upon ERS occurrence, PERK autophosphorylation activates eEF2α, decreases the translation level of most mRNAs in the cytoplasm, inhibits protein synthesis, as well as selectively promotes preferential translation of UPR-dependent genes such as ATF4 [37]. Steatosis is the first stage of NAFLD and is characterized by the ectopic accumulation of triglycerides in hepatocytes [38]. Since a large amount of lipid synthesis occurs in the smooth endoplasmic reticulum, the endoplasmic reticulum stress response plays an important role in the pathogenesis of steatosis [39]. The PERK-eIF2α-ATF4 arm was reported to regulate lipogenesis and steatosis [6]. PERK deletion inhibited the sustained expression of FAS, ACL and SCD1 in immortalized murine embryonic fibroblasts [40]. Overexpression of ATF4 triggers liver steatosis in zebrafish [11], while silencing of ATF4 leads to a reduction of lipogenic genes, including PPAR-γ, SREBP-1, ACC and FAS, in the liver and adipose tissue of mice [9]. ATF4-null mice were protected from age-related and diet-induced obesity and steatosis [41, 42]. In the present study, RT-PCR and western blot results showed that the gene and protein expression levels of PERK, p-PERK, eIF2α, p-eIF2α, ATF4 and ACC were significantly increased after administration of HFD feeding, and the abnormal expression of these genes and proteins were significantly reversed after the administration of HQT and FF intervention [18]. The results suggested that HQT intervention could alleviate endoplasmic reticulum stress-induced hepatic steatosis by inhibiting PERK signaling pathway-related mRNA and protein expression.

In addition, ATF6 plays an important role in the ERS process [43]. ATF6 is a type II transmembrane protein on the endoplasmic reticulum and is a member of the ATF/CREB family of transcription factors. When ERS occurs, activated ATF6 translocates to the Golgi apparatus, where it is cleaved by the resident proteases S1P and S2P, releasing a cytosolic fragment that migrates to the nucleus to regulate gene transcription [44]. Furthermore, ATF6 is primarily involved in cellular inflammation and apoptosis through translocation from the ER membrane to the nucleus [45, 46]. Under chronic conditions of endoplasmic reticulum stress, rather than alleviating the current stress, UPR is counterproductive and leads to key characteristics of progressive NASH, including inflammation and cell death [47]. It has been reported that ATF6α can activate NF-kB through phosphorylation of AKT, and the p65 subunit of NF-κB molecule can bind to DNA-specific sites and induce monocytes and macrophages to synthesize and release inflammatory factors CRP, TNF-α, IL-1β in large quantities, forming an inflammatory cascade response and causing liver injury [12]. Meanwhile, activated PERK can increase NF-κB activity by reducing the translation of IκB [48]. ATF6 and PERK/IRE1α arms appear to be essential for endoplasmic reticulum stress-induced NF-κB activation [49]. From the results of our study, we concluded that the expression of ATF6 protein and mRNA was significantly elevated in the HFD group compared with the Con group, whereas it was significantly down-regulated by the administration of HQT and FF treatments. In addition, the expression level of NF- κBp65 protein was also significantly reduced in the treatment group. In summary, HQT may interfere with PERK and ATF6 signaling pathway protein expression to reduce the intensity of ERS, attenuate the hepatocyte inflammatory response, and mitigate the progression of NAFLD.

## Conclusion

In summary, we have demonstrated that HQT is effective in reducing endoplasmic reticulum stress, lowering serum ALT, AST, TC and LDL-C levels, increasing HDL-C levels, and lowering inflammatory factors such as TNF-α, IL-6 and IL-1β levels. HQT may alleviate hepatic lipid accumulation and anti-inflammatory effects by downregulating GRP78, which inhibits PERK and ATF6 signaling pathways, thereby suppressing the expression of p-EIF2α, ACC and NF-κB-p65. Therefore, HQT can alleviate hepatic lipid accumulation and inflammatory response by inhibiting endoplasmic reticulum stress and can be used as a potential treatment for NAFLD.

### Supplementary Information

**Additional file 1.**

## Data Availability

The datasets used and/or analyzed during the current study available from the corresponding author on reasonable request.

## Abbreviations

ACC: Acetyl-coenzyme A-carboxylase
ALT: Alanine aminotransferase
AST: Aspartate aminotransferase
ATF4: Activating transcription factor 4
ATF6: Activating transcription factor 6
EIF2α: Eukaryotic translation initiation factor 2α
GRP78: Glucose regulating protein 78
HDL-C: High-density lipoprotein cholesterol
HFD: High fat diet
HQT: Hugan Qingzhi tablet
IL-1β: Interleukin-1β
IL-6: Interleukin-6
LDL-C: Low-density lipoprotein-cholesterol
NAFLD: Nonalcoholic fatty liver disease
NASH: Nonalcoholic steatohepatitis
NF-κB-p65: Nuclear factor-kappa B-p65
PERK: Protein kinase RNA-like endoplasmic reticulum kinase
TC: Cholesterol
TG: Triglyceride
TNF-α: Tumor necrosis factor α
