# Supplementary material for: Hugan Qingzhi tablets attenuates endoplasmic reticulum stress in nonalcoholic fatty liver disease rats by regulating PERK and ATF6 pathways
Source: BMC Complement Med Ther. 2024 Jan 12;24:36. doi: 10.1186/s12906-024-04336-1 (PMC10785447; doi:10.1186/s12906-024-04336-1)

Supplemental Files for：

Hugan Qingzhi tablets attenuates endoplasmic reticulum stress in nonalcoholic fatty liver disease rats by regulating PERK and ATF6 pathways

Miaoting Yang[1], Xiaorui Yao[2], Fan Xia[3], Shijian Xiang[3], Waijiao Tang[4], Benjie Zhou[3]*

1. Department of Pharmacy, People's Hospital of Longhua, Shenzhen, 518109, Guangdong, China.

2. Department of Pharmacy, Shantou Central Hospital, Shantou, 515041, Guangdong, China.

3. Department of Pharmacy, the Seventh Affiliated Hospital, Sun Yat-sen University, Shenzhen, 518107, PR China

4. Zhujiang Hospital, Southern Medical University, Guangzhou, 510282, Guangdong, China.

Address correspondence to:

Benjie Zhou: zhoubj23@mail.sysu.edu.cn; Tel.: +86 075581206186; Fax: +86 075581206166.

ORCID ID: https://orcid.org/0000-0001-5630-110X

E-mail addresses:

Miaoting Yang: 13392971213@163.com; Xiaorui Yao: 312578497@qq.com; Fan Xia: xiafan@sysush.com;

Shijian Xiang: xiangshj3@mail.sysu.edu.cn Waijiao Tang: tangwaijiao_2006@126.com

Supplementary Figure S1. The Western blot images corresponding to Fig.7a

- GRP78-full unedited gel for Figure 7a

GRP78-Repeat 1

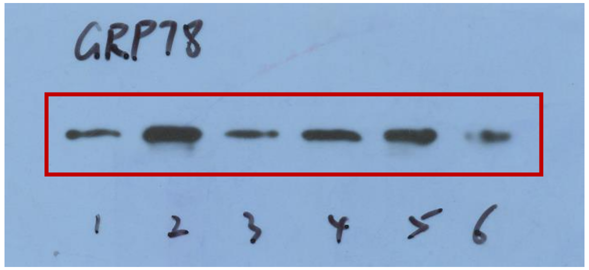

(ps: developed by film development)

GRP78-Repeat 2

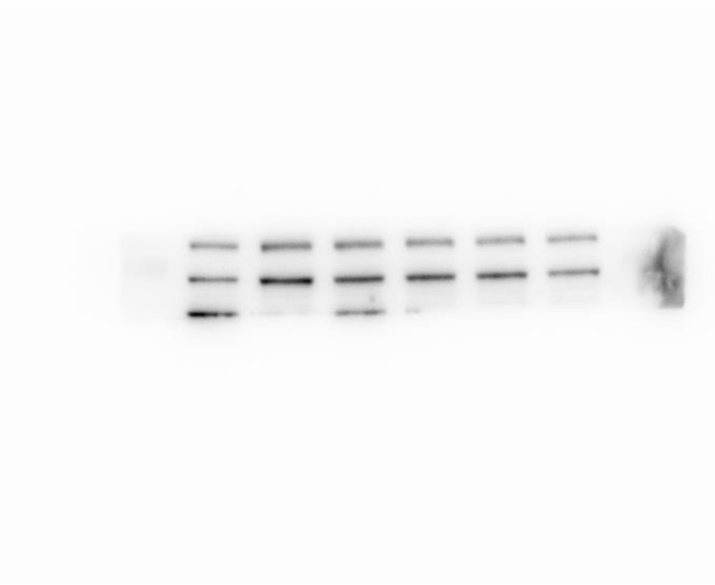

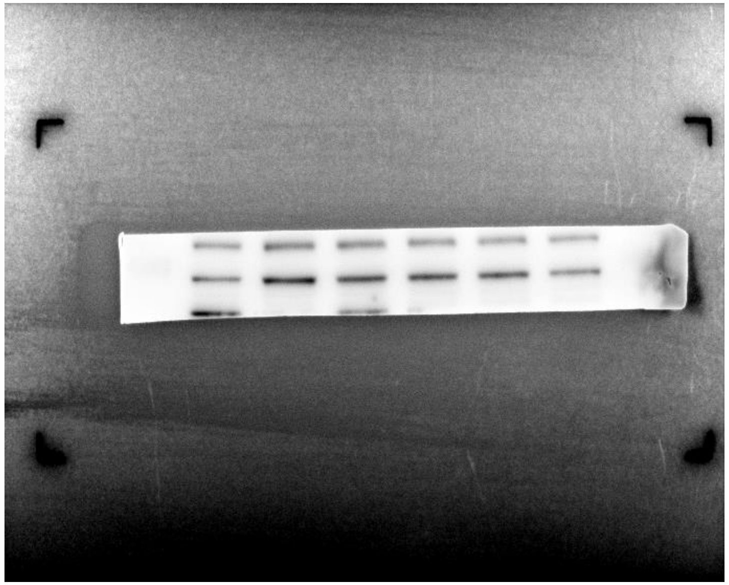

- **PERK-full unedited gel for Figure 7a**

PERK-Repeat 1

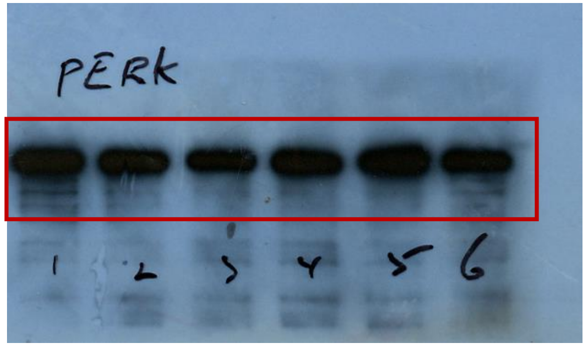

(ps: developed by film development)

PERK-Repeat 2

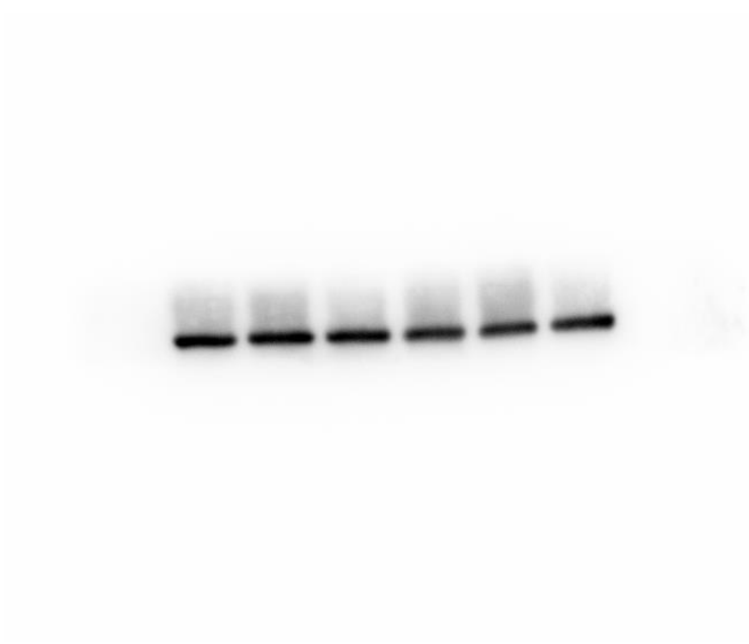

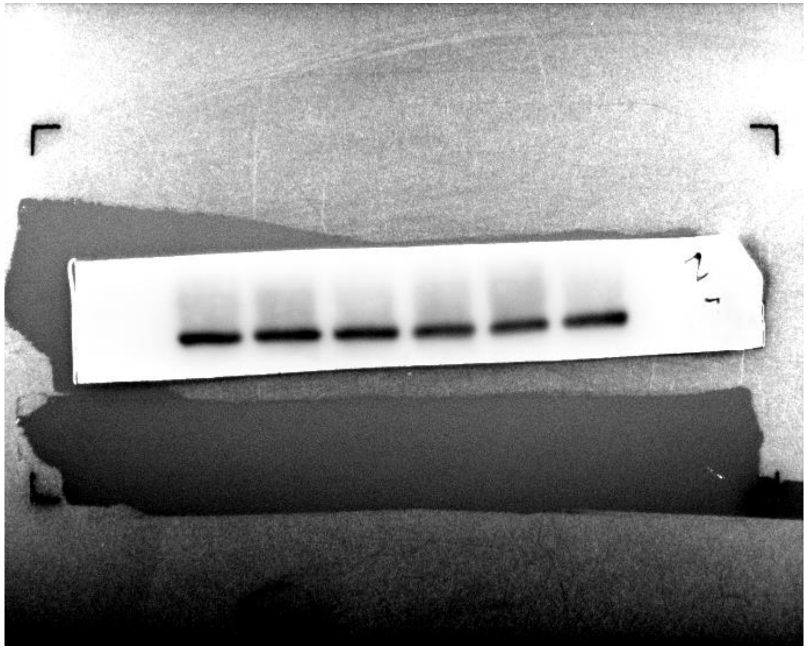

- **p-PERK-full unedited gel for Figure 7a**

  p-PERK-Repeat 1

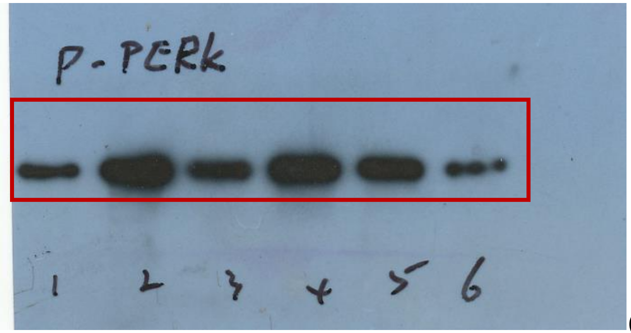

(ps: developed by film development)

p-PERK-Repeat 2

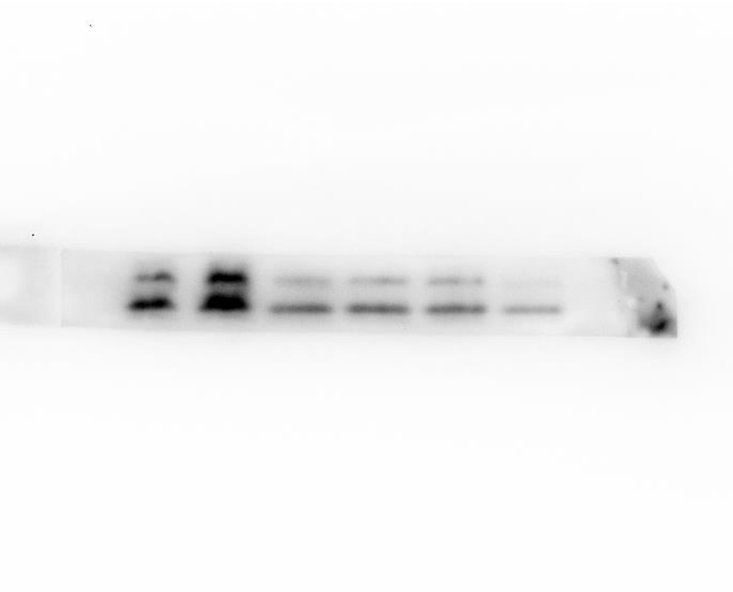

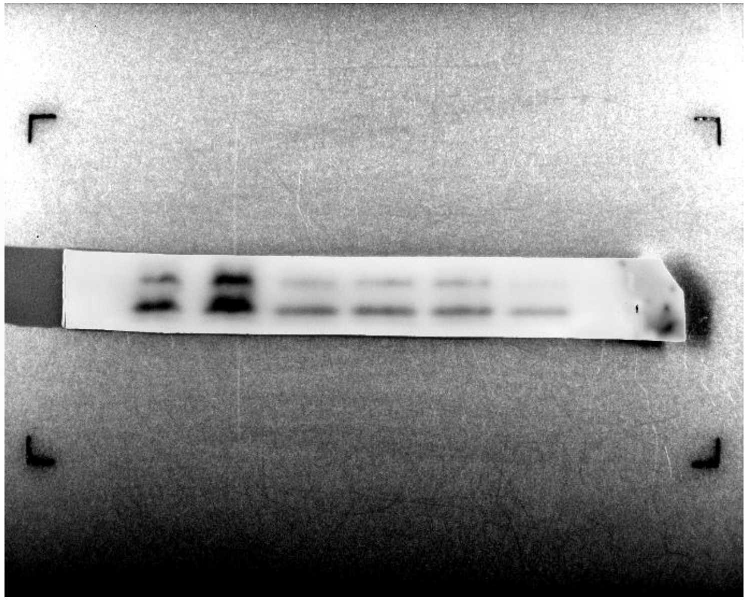

• **EIF2α-full unedited gel for Figure 7a**
EIF2α-Repeat1

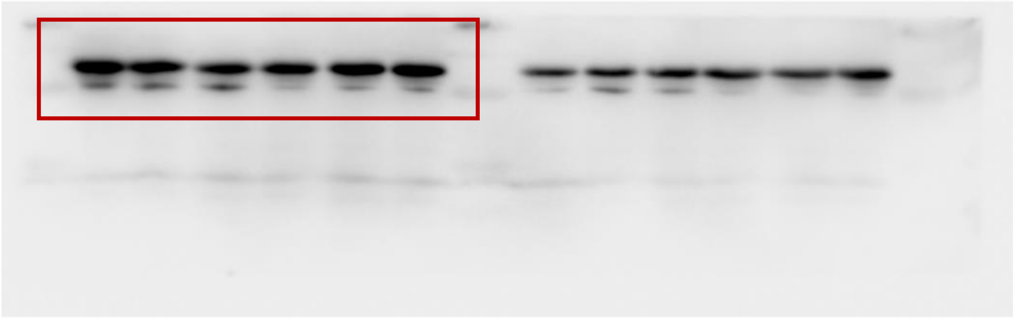

EIF2α-Repeat2

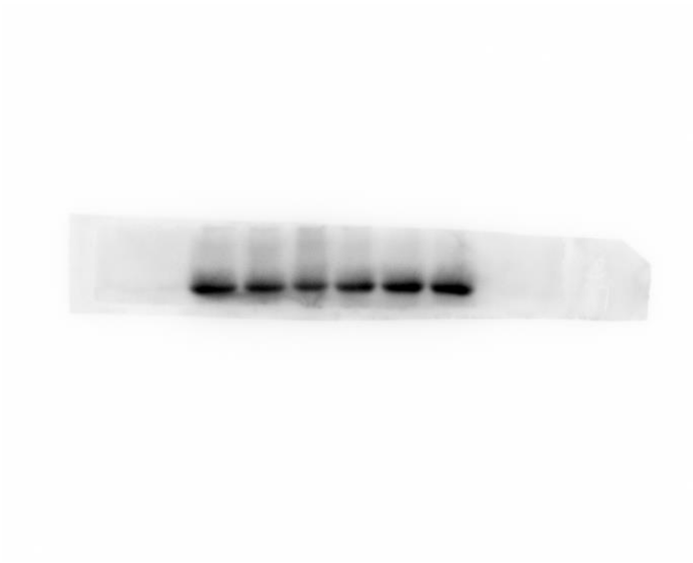

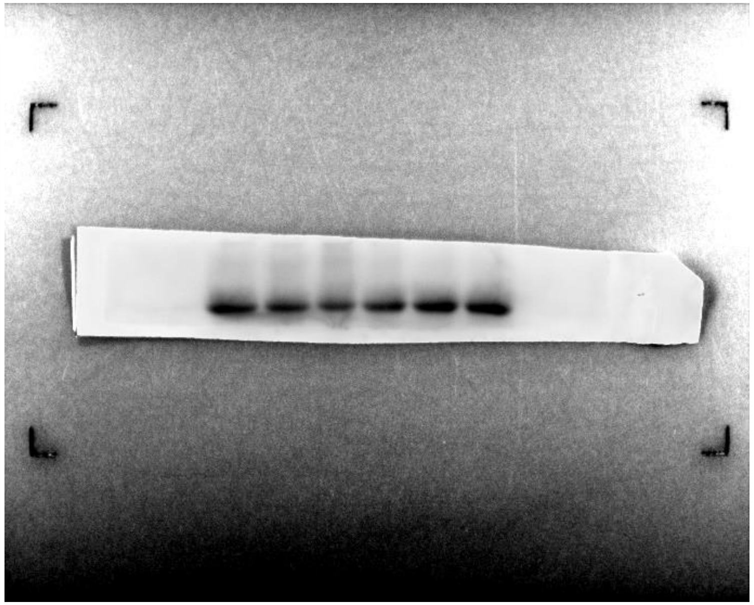

- **p-EIF2α-full unedited gel for Figure 7a**

p-EIF2α-Repeat1

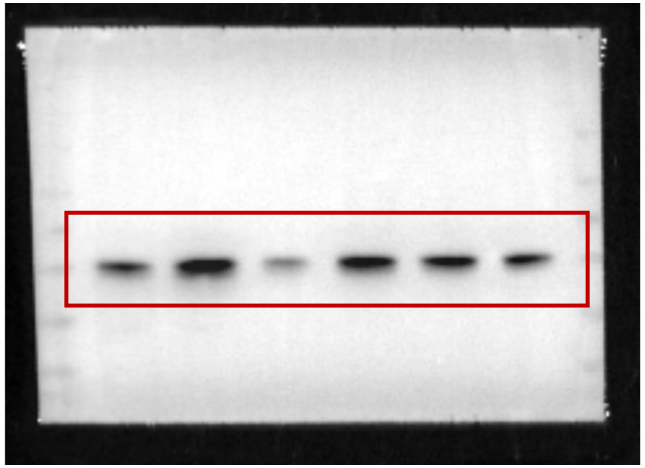

p-EIF2α-Repeat2

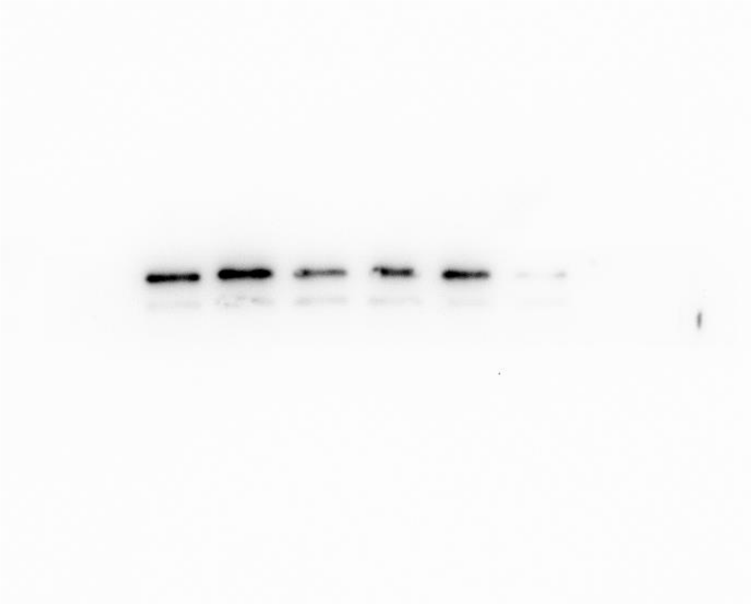

- **ATF4-full unedited gel for Figure 7a**

ATF4- Repeat 1

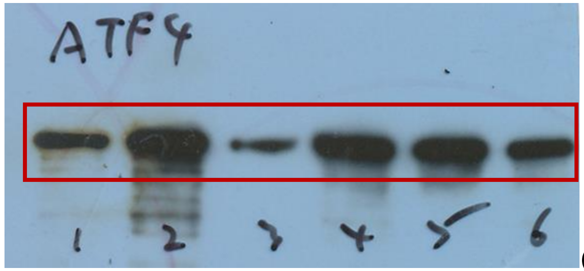

(ps: developed by film development)

ATF4- Repeat 2

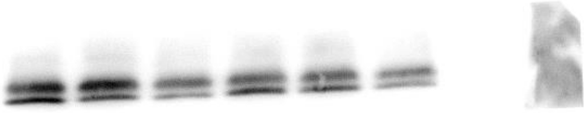

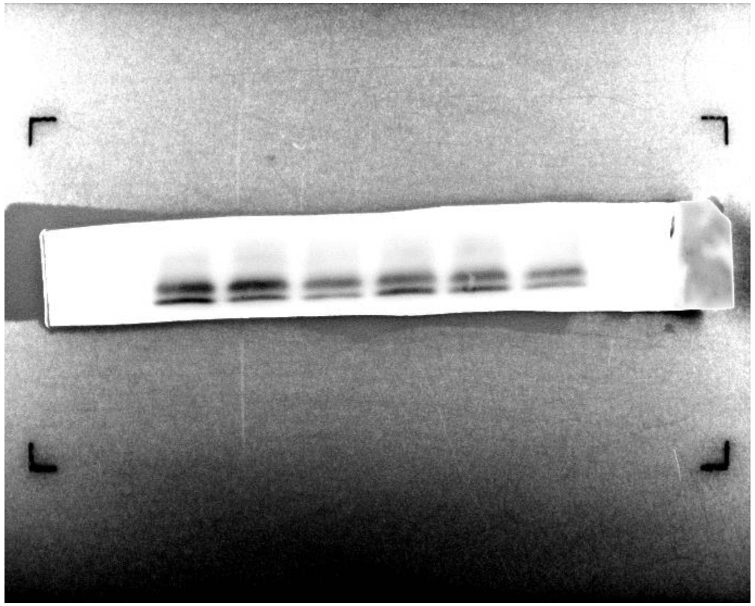

- **ACC -full unedited gel for Figure 7a**

ACC -Repeat 1

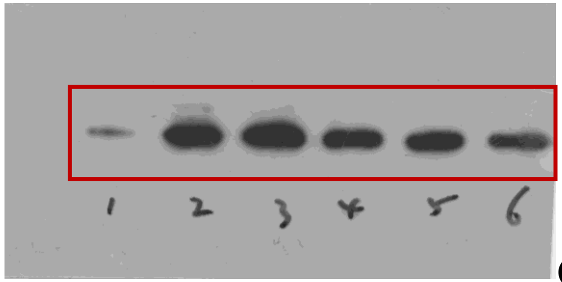

(ps: developed by film development)

ACC -Repeat 2

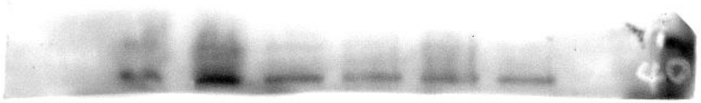

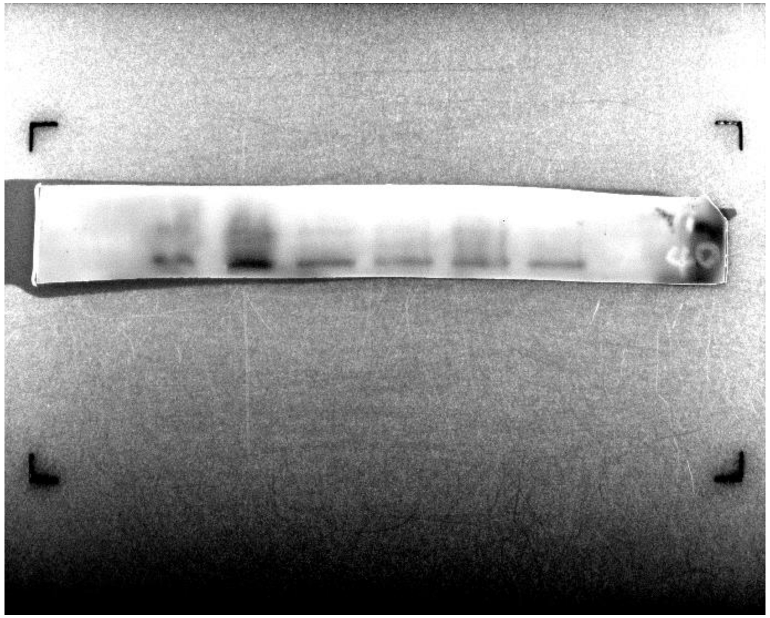

- **GAPDH -full unedited gel for Figure 7a：**

GAPDH - Repeat 1

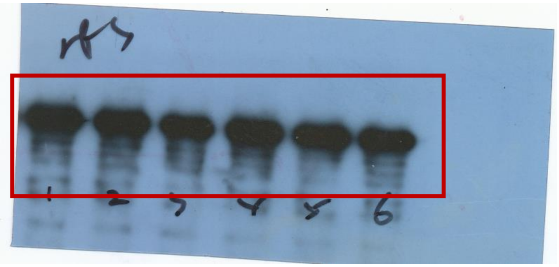

(ps: developed by film development)

GAPDH - Repeat 2

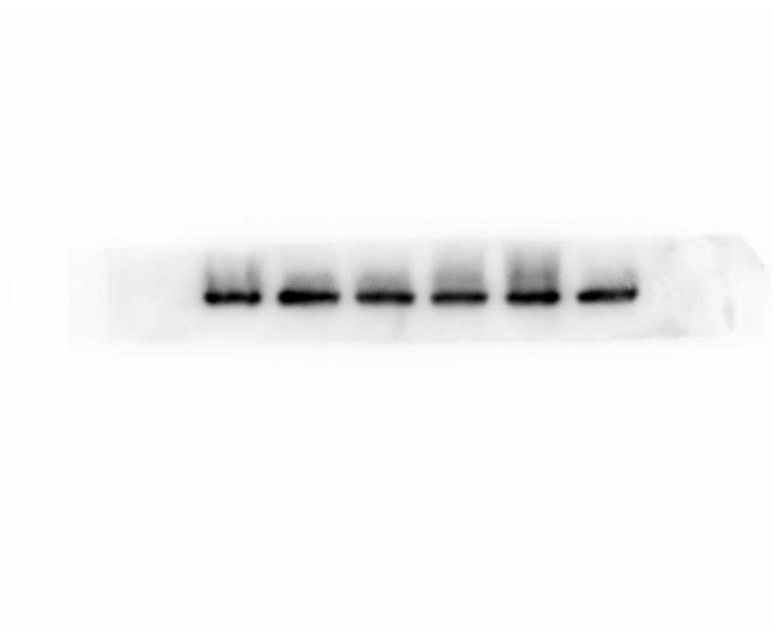

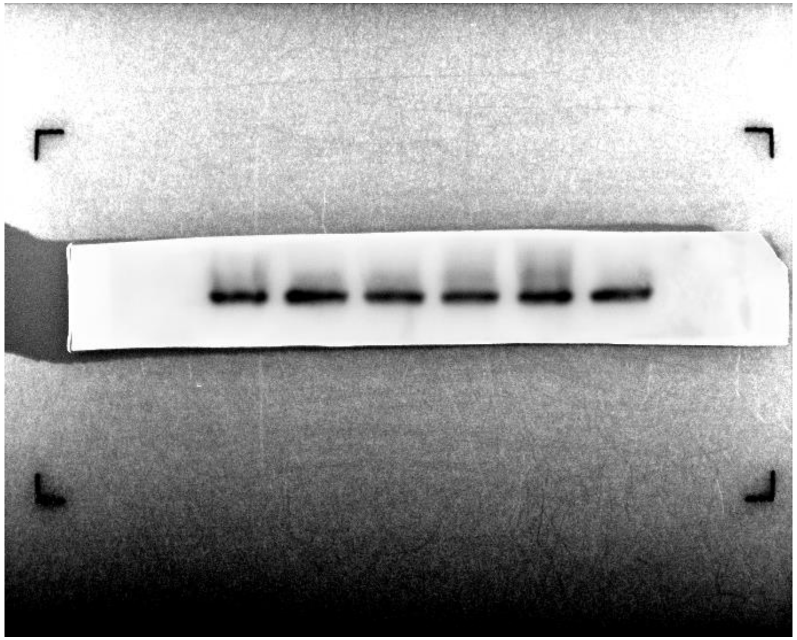

Supplementary Figure S2. The Western blot images corresponding to Fig.8a

- **ATF6-full unedited gel for Figure 8a:**

ATF6-Repeat 1

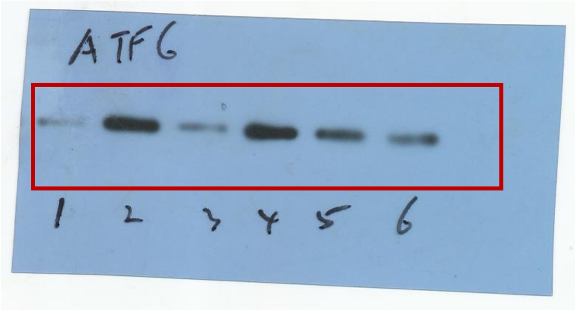

(ps: developed by film development)

ATF6-Repeat 2

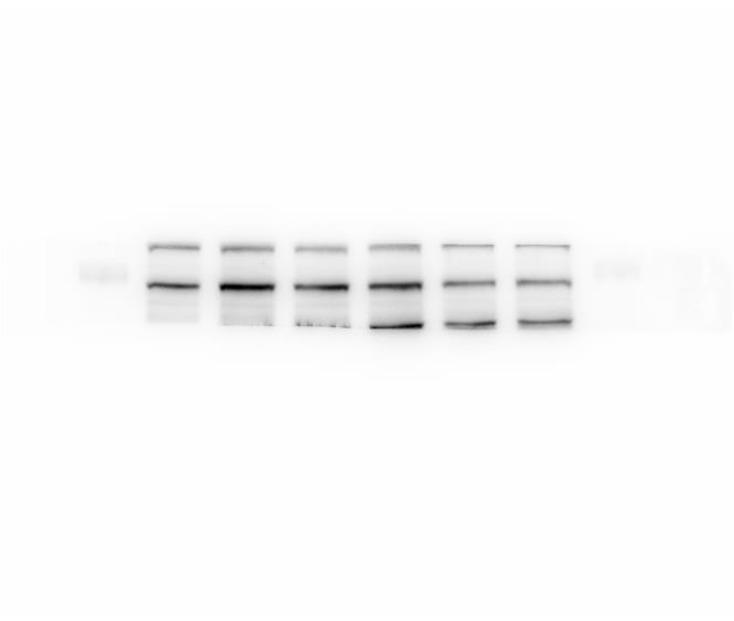

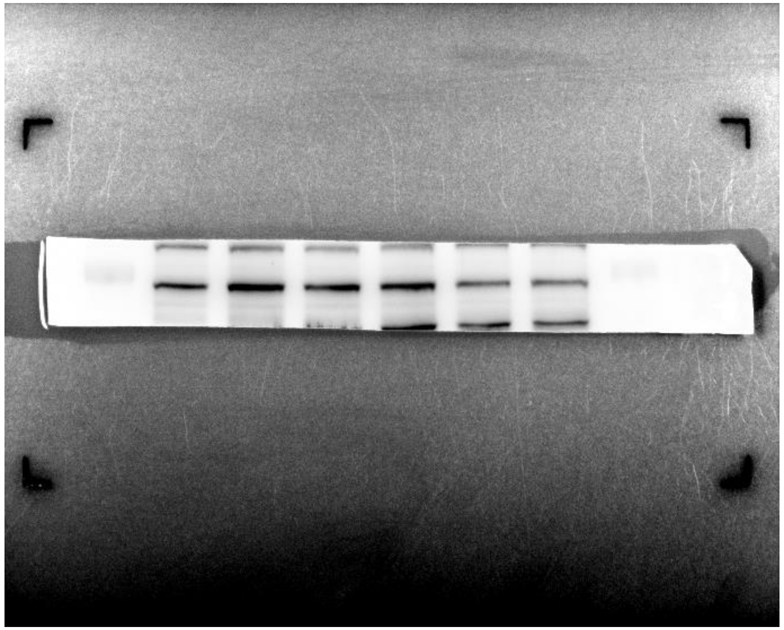

- **NF-κB-p65-full unedited gel for Figure 8a：**
  NF-κB-p65-Repeat 1

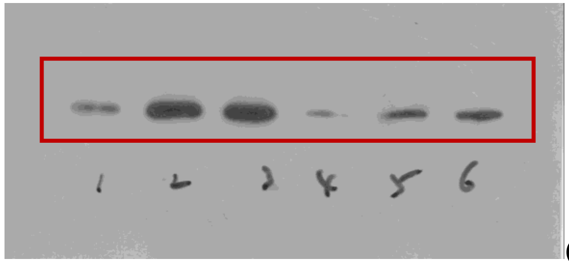**(ps: developed by film development)**

NF-κB-p65-Repeat 2

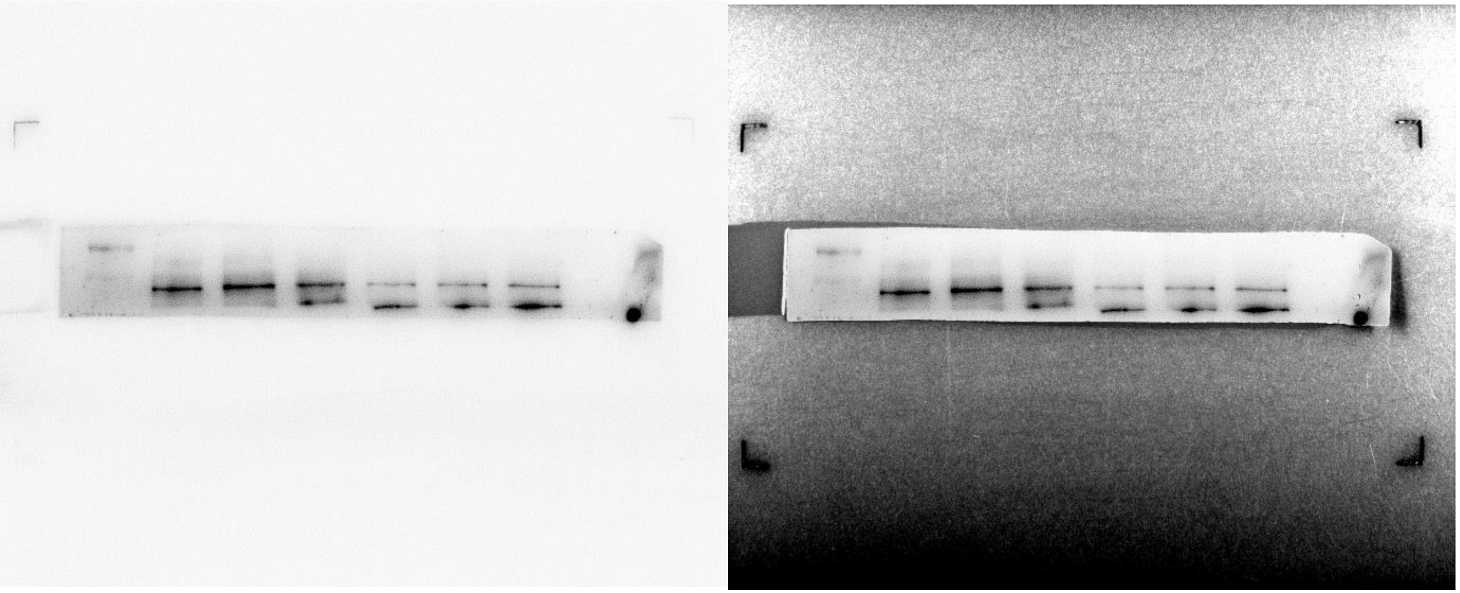

• **GAPDH-full unedited gel for Figure 8a：**
GAPDH-Repeat1

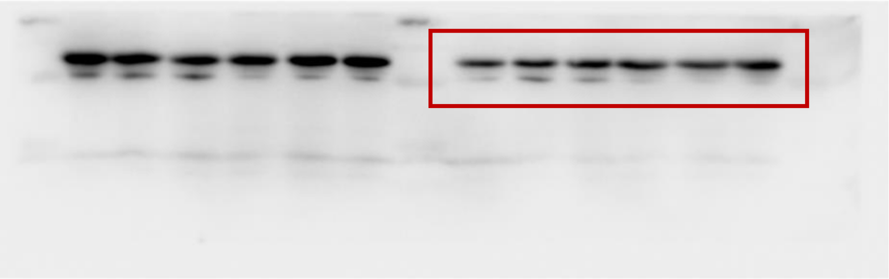

GAPDH-Repeat2

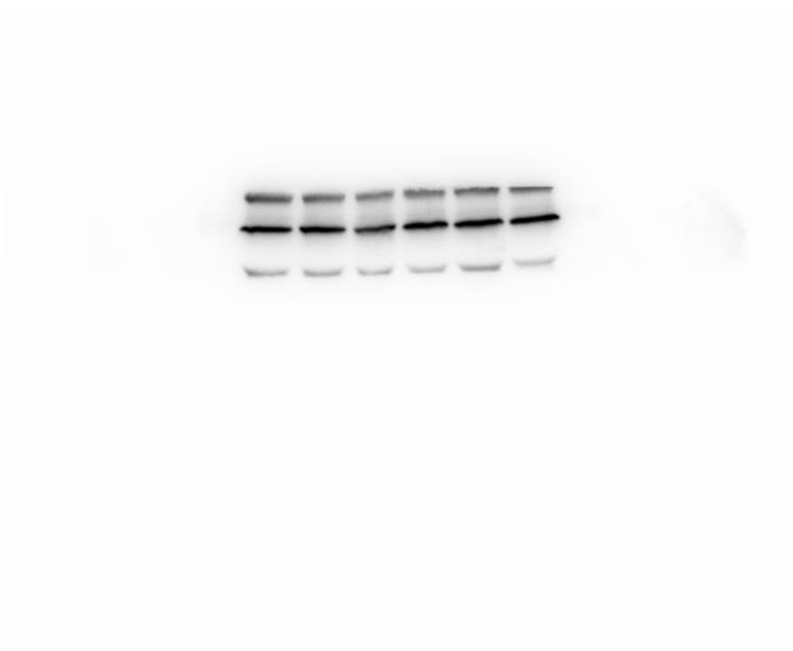

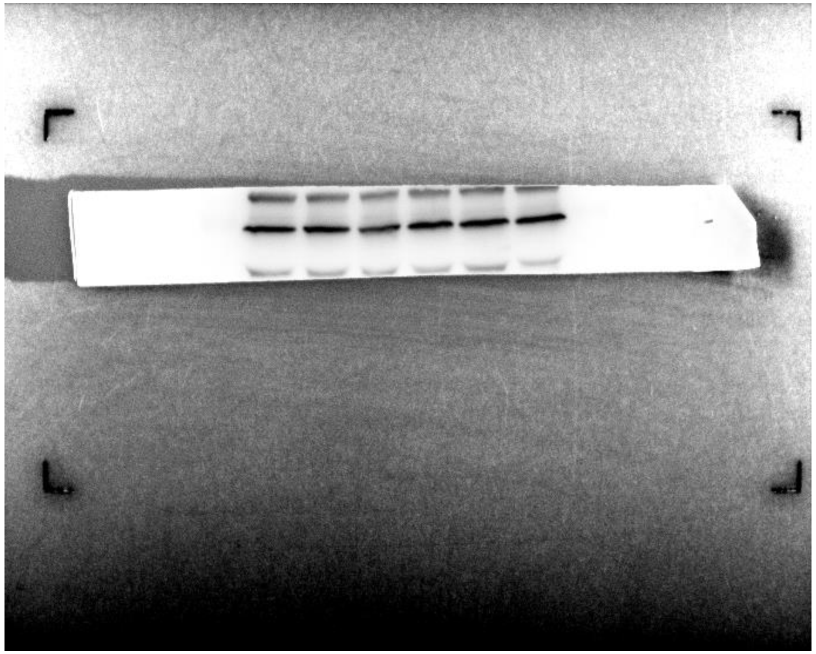

Supplement: Supplementary file 1 — Additional file 1. [file 12906_2024_4336_MOESM1_ESM.pdf]
